# Discovery of *GLO1* New Related Genes and Pathways by RNA-Seq on A2E-Stressed Retinal Epithelial Cells Could Improve Knowledge on Retinitis Pigmentosa

**DOI:** 10.3390/antiox9050416

**Published:** 2020-05-13

**Authors:** Luigi Donato, Concetta Scimone, Simona Alibrandi, Giacomo Nicocia, Carmela Rinaldi, Antonina Sidoti, Rosalia D’Angelo

**Affiliations:** 1 Department of Biomedical and Dental Sciences and Morphofunctional Imaging, Division of Medical Biotechnologies and Preventive Medicine, University of Messina, 98125 Messina, Italy; cscimone@unime.it (C.S.); simona.alibrandi@live.it (S.A.); crinaldi@unime.it (C.R.); rdangelo@unime.it (R.D.); 2 Department of Biomolecular strategies, genetics and avant-garde therapies, I.E.ME.S.T., 90139 Palermo, Italy; 3 Department of Chemical, Biological, Pharmaceutical and Environmental Sciences, University of Messina, 98125 Messina, Italy; 4 Department of Clinical and Experimental Medicine, University of Messina, 98125 Messina, Italy; giacomo.nicocia@unime.it

**Keywords:** *GLO1*, A2E, oxidative stress, RNA-Seq, retinitis pigmentosa

## Abstract

Endogenous antioxidants protect cells from reactive oxygen species (ROS)-related deleterious effects, and an imbalance in the oxidant/antioxidant systems generates oxidative stress. Glyoxalase 1 (GLO1) is a ubiquitous cellular enzyme involved in detoxification of methylglyoxal (MG), a cytotoxic byproduct of glycolysis whose excess can produce oxidative stress. In retinitis pigmentosa, one of the most diffuse cause of blindness, oxidative damage leads to photoreceptor death. To clarify the role of *GLO1* in retinitis pigmentosa onset and progression, we treated human retinal pigment epithelium cells by the oxidant agent A2E. Transcriptome profiles between treated and untreated cells were performed by RNA-Seq, considering two time points (3 and 6 h), after the basal one. The exposure to A2E highlighted significant expression differences and splicing events in 370 *GLO1* first-neighbor genes, and 23 of them emerged from pathway clustered analysis as main candidates to be associated with retinitis pigmentosa. Such a hypothesis was corroborated by the involvement of previously analyzed genes in specific cellular activities related to oxidative stress, such as glyoxylate and dicarboxylate metabolism, glycolysis, axo-dendritic transport, lipoprotein activity and metabolism, SUMOylation and retrograde transport at the trans-Golgi network. Our findings could be the starting point to explore unclear molecular mechanisms involved in retinitis pigmentosa etiopathogenesis.

## 1. Introduction

In recent years, extensive evidence has associated oxidative stress to the pathophysiology of many human diseases, and this phenomenon has now become a key focus of translational research. Oxidative stress was defined as an unbalanced level of reactive oxygen species (ROS) and intrinsic antioxidant defenses [1]. ROS act as second messengers in numerous intracellular signaling cascades, helping to maintain cellular homeostasis [2]. However, their excess can cause undiscriminating damage to biological molecules, inducing loss of function and even cell death [3]. In addition to ROS, high levels of advanced glycation end products (AGEs), heterogeneous molecules produced from nonenzymatic protein glycation reactions, are also usually increased in dysregulated redox balances and oxidative stress conditions, and determine hyperinflammation and permanent tissue damage [4]. 

Conventional products of metabolism such as α-oxoaldehydes, methylglyoxal (MG), glyoxal (GO) and 3-deoxyglucosone (3-DG), levels of which are increased in cells undergoing hyperglycemic metabolism, represent the principal source of intra- and extracellular AGEs [5]. AGEs exert their injuring effects by direct glycation of intracellular proteins and lipids, by the activation of cell signaling pathways through their binding to cellular receptors and modulation of gene expression, and by vessel elasticity reduction following atypical crosslinking of AGEs to stable and long-lived proteins of extracellular matrix [6]. 

Thus, the activity of antioxidant systems is crucial to finely regulate ROS and AGE levels. One of the major complexes involved in this protective role is the glyoxalase system, made of Glo1 (glyoxalase I), Glo2 (glyoxalase II) and glutathione as cofactors [7]. The main substrate of the glyoxalase system is MG, which reacts with proteins, especially with arginine residues, to form the AGE MG-H1 (MG-derived hydroimidazolone, isomer 1 or Nδ-(5-hydro-5-methyl-4-imidazolon-2-yl)ornithine) [8]. Other enzymes of the antioxidant system such as glutathione peroxidase and reductase can also be affected by methylglyoxal, leading to elevated ROS formation [9]. In the glyoxalase system, the key and limiting enzyme is GLO1, which is ubiquitous, cytosolic, and active in its dimeric form [10]. *GLO1* mutations have been linked to several human diseases, but very little is known about the influence of this enzyme in eye-related disorders, especially about genetic ones such as retinitis pigmentosa (RP) [11]. 

Retinitis pigmentosa (OMIM #600105) includes a wide-ranging group of inherited eye pathologies characterized by progressive vision loss, affecting about 1/4000 people in the United States, and 1-5/10,000 in Italy, even if several forms are significantly rarer [12,13]. RP progression rate and age of onset depend on numerous factors, especially genetic transmission pattern [14]. The disorder may be inherited as an autosomal dominant (25%), autosomal recessive (39%), or X-linked recessive (4%) trait; mitochondrial inheritance, digenism and uniparental isodisomy have also been described [15]. Today, mutations in more than 80 genes have been associated to RP [16]. 

The cytological targets of these disorders usually regard photoreceptors, especially rods responsible of daylight and central vision, and retinal pigment epithelium (RPE), although other retinal cytotypes are not excluded [17]. Originally, only a trophic function was imagined for RPE cells. Nowadays, it is well-known that the RPE consists of a monolayer of neural-crista-derived pigmented epithelial cells which interacts with the outer segments of the photoreceptors (POS) on the apical side and with Bruch’s membrane and choriocapillaris on the basolateral one [18]. RPE guarantees lots of vital functions for photoreceptors, such as absorption of light, epithelial bidirectional transport, spatial buffering of ions, visual cycle regulation, phagocytosis of photoreceptor outer segments (POS), secretion of trophic factors and signaling molecules, and support of the immune privilege of the inner eye (disconnected from the immune system of the blood stream) [19]. However, the most fascinating activity exerted by RPE is represented by oxidative stress protection [20,21]. Numerous studies confirmed the presence of high levels of ROS and AGEs in RPE, which are able to alter transduction pathways and genic expression [22]. Despite this, oxidative stress mechanisms in RP development is not enough clear. 

To better understand how GLO1 activity is related to high ROS and AGE concentration, influencing RP onset and progress, we performed a comparison of transcriptome profiles among human RPE cells exposed to the oxidant agent N-retinylidene-N-retinylethanolamine (A2E) and untreated cells. Initial dysfunction and degeneration of RPE cells generally determine the accumulation of metabolic waste between choroidal and RPE layers, called drusen. Drusen presents mixtures of highly photosensitive lipofuscin, involved in the first stages of photosensitive reactions due to the generation of singlet oxygen and the superoxide anion. A2E is one of the most abundant components of drusen, and direct transmittal of light to the retina via drusen implies its cleavage at the pyridinium ring, inducing oxidative stress [23]. The main purpose of our study was the identification of the complex gene network in which *GLO1* might be involved, together with the related pathways and the detection of differential expression of genes most likely linked to RP development.

## 2. Materials and Methods

### 2.1. Cell Culture

Human Retinal Pigment Epithelial Cells (H-RPE, Clonetics™, Lonza, Walkersville, MD, USA) were cultivated, and then grown for 24 h to reach confluence, as previously described [24]. Subsequently, a group of cells was treated with A2E 20 μM for 3 and 6 h before rinsing with medium, while a control group was incubated without the oxidant agent. Finally, confluent cultures were transferred to PBS-CMG and, then, subjected to blue light delivered by a tungsten halogen source (470 ± 20 nm; 0.4 mW/mm^2^) for 30 min, in order to induce phototoxicity of A2E, and incubated at 37 °C for 24 h. Each group of cells had three biological replicates.

### 2.2. MTT Assay

The mitochondrial-dependent reduction of methylthiazolyldiphenyl-tetrazolium bromide (MTT) (Sigma-Aldrich, St. Louis, MO, USA) to formazan insoluble crystals was realized to analyze cell viability, following a protocol already described [24]. Finally, a Dynatech microplate reader permitted evaluation of the absorbance at 570 nm, and results were expressed as a percentage of viable cells compared with control conditions in the absence of A2E. Multiple *t*-tests were performed for statistical comparison (*p*-value < 0.05), considering 3 independent experiments, each one characterized by 3 replicates.

### 2.3. Total RNA Isolation and RNA-Seq Profiling

Total RNA was extracted, checked for degradation and contamination, and quantified as previously reported [24]. The RNA-seq samples were divided in 3 factor groups, consisting of human RPE cells before A2E treatment and at the different time points of 3 and 6 h, respectively. Each group was biologically replicated three times, for a total of 9 samples. Both 3 and 6 h time points were chosen on the basis of previous experiments realized by our research group (unpublished data), confirmed by results obtained from the MTT assay in this work. Such outcomes showed that in wider time intervals, the death rate of oxidative stressed cells might be so high as to invalidate the following expression analysis. Libraries were generated using 1 µg of total RNA by the TruSeq Stranded Total RNA Sample Prep Kit with Ribo-Zero H/M/R (Illumina, San Diego, CA, USA), following the manufacturer’s protocols. The last step involved the sequencing of the libraries on an HiSeq 2500 Sequencer (Illumina, San Diego, CA, USA), using the HiSeq SBS Kit v4 (Illumina, San Diego, CA, USA).

### 2.4. Data Analysis

Obtained raw reads were quality trimmed and then mapped against the hg38 reference genome and Ensembl RNA database v.99 (EMBL-EBI, Hinxton, Cambridgeshire, UK) by Qiagen CLC Genomics Workbench v.20.0.2 (Qiagen, Hilden, Germany) [25], following criteria adopted by Donato et al. [24]. Aligned reads were quantified by a mapping-dependent expectation-maximization (EM) algorithm [26], and the transcript per million reads (TPM) values were then computed after normalization by the trimmed mean of M-values (TMM) method [27]. Differential expression analysis was realized using the Limma R package [28], setting the contrast groups as 0 (untreated) versus 3 h (treated), 0 (untreated) versus 6 h (treated), 3 versus 6 h (both treated) and ((0 h.untreated+3 h.treated)/2)-((0 h.untreated+6 h.treated)/2). The latter resulted from a multiple group mean comparison, that permitted the indirect detection of the differences in expression level induced by the whole period of treatment, hereafter called “Due to Time” effect. For differentially expressed genes, the log_2_ fold change (log_2_FC) of their abundance was calculated based on contrast groups, and significance of expression changes was determined using the *t*-test [29]. Benjamini and Hochberg (BH) post hoc test was then applied to correct false discovery rate (FDR) on *p*-values obtained by multiple testing [30]. The genes uniquely identified in the RPE cells, presenting at least 3 unique gene reads, lower than two-fold (log_2_FC < −1, down-regulated) or greater than two-fold (log_2_FC > 1, up-regulated) changes in expression, and with BH–adjusted p-values lower than 0.05, were chosen for functional classification. 

### 2.5. Quantitative RT-PCR (qRT-PCR) Validation

To assess the reliability of RNA-Seq data, ten of most dysregulated genes from the whole RNA-Seq analysis were selected to be validated by quantitative Real-Time polymerase chain reaction (qRT-PCR). Reverse transcription was performed with a GoScript™ Reverse Transcription System (Promega, Madison, WI, USA), according to manufacturer’s protocol. The qRT-PCR was, then, applied on obtained cDNA in the ABI 7500 fast sequence detection system (Applied Biosystems, Foster City, CA, USA), using the BRYT-Green based PCR reaction, as previously performed [24]. Each reaction was replicate 6 times, considering all analyzed time points (18 samples), and the average threshold cycle (Ct) was calculated for each replicate. Gene expression was normalized to the expression level of most stable reference gene, identified as combination of GeNorm [31], BestKeeper [32], Delta Ct [33] and NormFinder [34] algorithm results. The relative gene expression was then estimated using the 2^−ΔΔCt^ method, and the results were shown as the mean ± SEM (Standard Error of Mean). The analysis of variance between groups (ANOVA), corrected by Bonferroni post hoc test, permitted the assessment of the statistical significance. Lastly, a linear regression analysis was carried out to check the correlation of fold change ratios between RNA-Seq and qRT-PCR. The whole statistical analyses were executed using IBM SPSS 26.0 software (https://www.ibm.com/analytics/us/en/technology/spss/). Corrected *p*-values < 0.05 were considered as statistically significant.

### 2.6. Pathway Analysis

GO term enrichment analysis for the most dysregulated genes was performed using the GeneMANIA (v. 3.5.2) (University of Toronto, Toronto, Canada), ClusterMaker2 (v. 1.3.1) (University of California, CA, USA), ClueGO (v. 2.5.6) (INSERM, Paris, France) [35] and CluePedia (v. 1.5.6) (INSERM, Paris, France) [36] plugins in Cytoscape (ver. 3.7.2) (National Institute of General Medical Sciences, Bethesda, MD, USA) [37]. GeneMANIA has been set to find the top 20 related genes and at most attributes using automatic weighting. ClusterMaker2 performed a clustering based on BestNeighbor Filter, set with the Proportion of node edges in cluster = 0.5. ClueGO options have been set as follow: CLINVAR, GO (Biological Process, Cellular Component, Molecular Function and Immune System Process), INTERPRO, KEGG, REACTOME (Pathways and Reactions), WIKIPATHWAYS and CORUM 3.0 as selected ontologies; GO Tree Interval Min Level = 3 and Max Level = 8; GO Term/Pathway Selection Min # Genes = 3 and % Genes = 4.000; GO Term/Pathway Network Connectivity (Kappa Score) = 0.4; Statistics Options set on Enrichment/Depletion (Two-Sided hypergeometric test), with pV correction = Bonferroni step-down. CluePedia was used following default settings. Finally, only GO terms with *p* < 0.01 were selected. 

## 3. Results

### 3.1. A2E Treatment Highlighted a Significant Negative Effect on RPE Cell Viability

**Figure 1:**
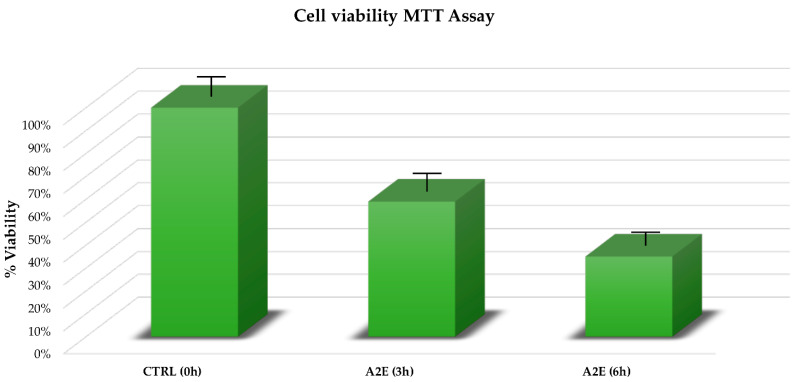
Cell viability from methylthiazolyldiphenyl-tetrazolium bromide (MTT) assay. Retinal pigment epithelium (RPE) cell viability percentage is expressed as mean of replicates ± standard error of mean, considering 3 replicates for each independent experiment (*n* = 3). Multiple *t*-tests were performed for statistical comparisons (*p*-value < 0.05). Results were estimated at both treatment considered time points (3 and 6 h) compared to the time zero untreated group.

The MTT assay showed a relevant impact of A2E treatment on RPE cell survival in a time-dependent manner. In contrast to the control group, the viability of treated RPE cells was significantly decreased (*p* < 0.05), especially after 6 h from treatment (Figure 1). 

### 3.2. A2E Treatment Highlighted GLO1 Down-Regulation, as Well as the Most of Its Related Genes

**Figure 2:**
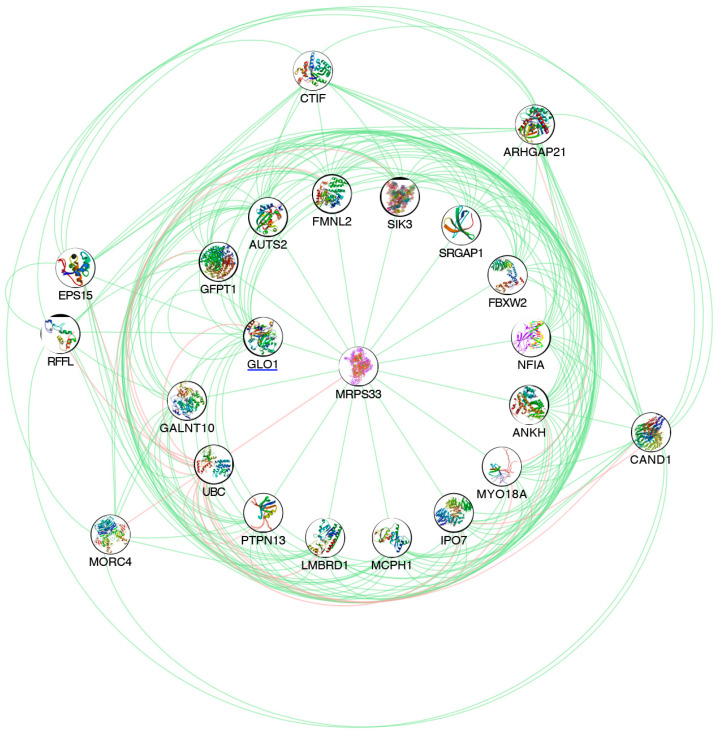
Pathway analysis of GLO1 and its best neighbors. The represented network highlights GLO1 and its best neighbors, emerged from GENEMANIA pathway analysis. Green edges indicate genetic interactions. Pink edges indicate physical interactions.

The RNA sequencing globally generated about 100 million quality reads (mean mapping quality = 29) and with a percentage of ~70% uniquely mapped. A total of 58,243 differentially expressed genes (DEGs) were detected out of 59,661 reference counterparts, considering the whole human genome annotations. All previous mapping statistics were based on average values calculated for all three replicates in each time point. Among all detected DEGs, 24,465 showed expression alterations in evaluated time points (Figure S1), and 370 of them were highly correlated to *GLO1*, as an output of GENEMANIA analysis. In detail, 244 were down-regulated, with the lowest expression value reached by *PIP5K1B* (log_2_FC = −2.207, *p*-value = 0.000), and 126 were up-regulated, with the highest value shown by *ARID5A* (log_2_FC = 1.918, *p*-value = 0.000) (Table S1). The subsequent clustering by ClusterMaker2 shed light on a particular group of genes, the best neighbors of *GLO1*, showing the same expression trend (Figure 2). Of this cluster consisted of 23 genes, 20 were globally down-expressed and only 3 were over-expressed. In detail, the lowest expression value was shown precisely by *GLO1* (log_2_FC = −1.088, *p*-value = 0.000), while the highest expression change resulted from *ANKH* (log_2_FC = 0.564, *p*-value = 8.656E-05) (Table 1).

### 3.3. qRT-PCR Validation

**Figure 3:**
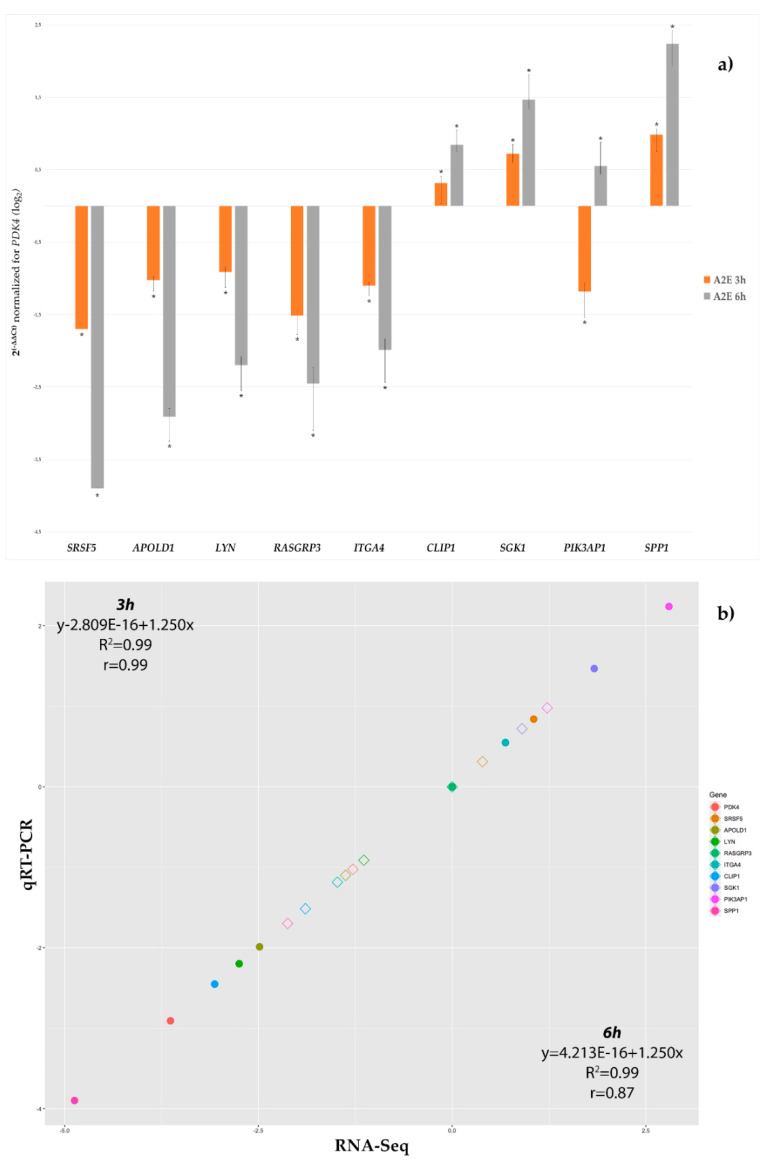
qRT-PCR validation of the ten most differentially expressed genes. (**a**) Histograms show the mean expression values (n° of replicate for each group = 6) of nine chosen differentially expressed genes produced by qRT-PCR experiments, resulting after application of the 2^−ΔΔCt^ method, normalized to the best stable gene RASGRP3 (not shown) and control group. Computed results were statistically significant (ANOVA Bonferroni-corrected *p*-values < 0.01). (**b**) Correlation plot between RNA-Seq log_2_FC and qRT-PCR log_2_FC data, as mean of all considered replicates, confirmed the RNA-Seq result validity. Empty Diamond = RNA-Seq log_2_FC value for that gene at 3 h (3 h vs. basal time). Full circle = qRT-PCR log_2_FC value for that gene at 6 h (6 h vs. basal time).

To confirm the reliability of DEGs identified by deep sequencing, a total of ten genes were chosen among most the dysregulated and pathway-related to *GLO1*, for confirmation in a biologically independent experiment using qRT-PCR. qRT-PCR analysis showed that the expression trends of the chosen genes matched with those observed by the RNA-Seq (Table 2 and Figure 3), although there were some little differences in the degree of the changes. The analysis of variance (ANOVA) method, conducted to compare the means among multiple groups, highlighted high significance (*p*-values < 0.05). 

### 3.4. Glycolysis and UPR Resulted the Most GLO1-Related Dysregulated Pathways Impaired by Induced Oxidative Stress

More than 60 clustered pathways resulted from enrichment of 370 *GLO1*-related genes by Cytoscape and its plugins ClueGO and CluePedia (Table S2). Among them, 14 pathways showed significant associations with the 22 *GLO1* most related neighbor genes (Table 3). These pathways are related to glycolysis and the unfolded protein response (UPR). As evidenced by GO, KEGG and Reactome databases in particular, cited pathways with high significance (Bonferroni step-down corrected group p-values near zero) were—“Asparagine N-linked glycosylation” (*p*-value = 1.179 × 10^−10^), “Clathrin recruits PIK3C2A” (*p*-value = 0.003), “Glycolysis” (*p*-value = 5.947 × 10^−26^), “Glyoxylate and dicarboxylate metabolism” (*p*-value = 8.247 × 10^−4^), “Golgi vesicle budding” (*p*-value = 1.076 × 10^−8^), “Rho GTPase cycle” (*p*-value = 6.752 × 10^−5^), “SMURFs ubiquitinate RUNX3” (*p*-value = 9.609 × 10^−19^), “Synthesis of PIPs at the plasma membrane” (*p*-value = 6.504 × 10^−6^), “Ubiquitin mediated proteolysis” (*p*-value = 9.683 × 10^−5^), “Unfolded Protein Response” (*p*-value = 0.003), “Chromosomal region” (*p*-value = 1.072 × 10^−27^), “Establishment of mitotic spindle orientation” (*p*-value = 0.002), “Regulation of lamellipodium organization” (*p*-value = 0.003) and “Translational termination” (*p*-value = 0.003). Moreover, nine of twenty-two *GLO1* most related neighbor genes were not significantly clustered, but individually share important pathways with *GLO1* (Table 3). Such genes belong to transcription factors (*MORC4* and *NFIA*), ubiquitin protein ligase activity (*FBXW2* and *RFLL*), translation initiation (*CTIF*), nuclear import of proteins (*IPO7*), inorganic pyrophosphate (PPi) transport regulation (*ANKH*), positive regulation of mTOR signaling (*SIK3*) and catalysis of mucin-type oligosaccharides (*GALNT10*). Further details on clustered pathways and unclustered genes are available in Table S3.

## 4. Discussion

The development of eye-related dystrophies is based on both environmental and genetic features. A common pathogenic factor is the accumulation of AGEs which are the toxic byproducts of the nonenzymatic reaction of proteins, lipids and nucleic acids with reducing sugars [38]. Advanced glycation (or glycosylation) occurs in all cytotypes and regards the post-translational modification of glucose-derived dicarbonyl compounds and amino residues present in proteins, lipids and DNA [39]. In the retina, AGEs could promote vascular dysfunction by altering intra- and extracellular protein structure and by increasing inflammation and oxidative stress [40]. The retinal AGE deposition could determine an upregulation of vascular endothelial growth factor (VEGF) and a downregulation of pigment epithelium-derived factor (PEDF), as well as a serious disruption of the inner blood–retinal barrier (iBRB) [41]. Additionally, boosted advanced lipoxidation end-products (ALEs) accumulation was also detected in the outer retina. This portion is mainly rich in polyunsaturated fatty acids that are highly susceptible to lipid peroxidation, further contributing to retinal aging and diseases [42].

Intracellular AGEs are able to induce post-translational modifications of regulatory proteins, especially those in the proteasome, and can directly bind and impair mitochondrial proteins involved in the electron transport chain, inhibiting oxidative ATP production [43]. Intracellular AGE precursors such as methylglyoxal (MG) and glyoxal (GO) can also modify and inhibit the function of important enzymes such as Glyceraldehyde-3-Phosphate Dehydrogenase (GAPDH) and glyoxalase 1 (GLO1) [44]. Glyoxalase 1, in particular, is part of the endogenous detoxification system that converts toxic AGE-forming dicarbonyls to less reactive products [45]. In detail, GLO1 metabolizes MG and prevents MG-induced damage. An excess of MG inactivates antioxidant enzymes such as glutathione peroxidase and SOD enzymes, impairing degradation of MG and determining a positive feedback loop [46]. The activity of GLO1 gradually declines during aging induces the accumulation of AGE in tissues [47]. *GLO1* overexpression was already associated with protective effects against neuroglial and vascular lesions [48], and its mutations could be involved in cancer onset [49], as well as in several genetic pathologies like cerebral cavernous malformations (CCMs) [50] and retinitis pigmentosa [11]. Even if it is known that GLO1 defects could play a role in retinitis pigmentosa, contributing to the accumulation of AGEs in the retina, very little is known about molecular mechanisms and pathways by which GLO1 determines its deleterious effects.

In order to achieve this purpose, we applied next generation sequencing (NGS) technologies to investigate the whole transcriptome of RPE cells during a follow-up of two time points (3 and 6 h) after exposure to activated oxidant compound A2E. Oxidative stress is currently recognized as one of the most relevant biochemical pathways involved in RP etiopathogenesis, especially targeting the high metabolic demand of RPE cells. In addition to the down-expression of *GLO1*, we found several *GLO1*-related genes dysregulated in treated cells. The analysis of these genes shed light on candidate pathways, shared with *GLO1*, probably responsible for the cellular damage phenotype.

Among these pathways, the one related to cytoskeleton dynamics and RhoGTPases activation cascades needs to be mentioned. Cytoplasmic Activator of Transcription and Developmental Regulator AUTS2 activates the Rho family small GTPase Rac1, a key coordinator of actin polymerization and microtubule dynamics, controlling neuronal migration and neurite extension. The nuclear form of AUTS2 acts as a transcriptional activator of several target genes, in complex with the polycomb complex 1 (PRC1) [51]. The other two genes encoding for proteins acting as RhoGTPase regulators are Rho GTPase Activating Protein 21 (*ARHGAP21*) and Protein Tyrosine Phosphatase Non-Receptor Type 13 (*PTPN13*), both involved in the down-regulation of cell migration and proliferation, cell polarity, cell adhesion, stress fiber formation and cell differentiation. ARHGAP21 also plays a relevant role in Golgi regulation and positioning, intracellular trafficking and glucose homeostasis [52]. PTPN13, instead, is a tyrosine phosphatase which mediates phosphoinositide 3-kinase (PI3K) signaling trough dephosphorylation of Phosphoinositide-3-Kinase Regulatory Subunit 2 (PIK3R2) [53] and regulates negative apoptotic signaling [54], as the antagonist of RhoGTPase A, SLIT-ROBO Rho GTPase Activating Protein 1 (SRGAP1) [55]. The latter is also involved in the modulation of contractility during epithelial junction maturation [56], a role also played by Formin Like 2 (FMNL2), probably by regulating actin polymerization and organization of the cytoskeleton [57]. Thus, the global down-expression of these genes (the only up-regulated gene is *AUTS2*) could indicate the alteration of actin filament structure and activity, leading to impairment in cell polarity and adhesion, with the final result of an increase in RPE apoptosis.

Moreover, *FMNL2* expression reduction, already known to be involved in glaucoma [58], causes huge Golgi dispersal, malformations of vesicular organelles and defective anterograde transport from the Golgi to plasma membrane [57]. The same consequences could occur with down-expression of Ubiquitin C (*UBC*) [59], Myosin XVIIIA (*MYO18A*) and Epidermal Growth Factor Receptor Pathway Substrate 15 (*EPS15*), and with over-expression of ANKH Inorganic Pyrophosphate Transport Regulator *(ANKH*). In particular, MYO18A is also involved in intracellular transport processes, in retrograde treadmilling of actin and in its transport from focal adhesions to the leading edge [60]. ANKH regulates the transmembrane efflux of ATP and the trans-Golgi network trafficking, as well as endocytosis [61]. The latter process is also modulated by *EPS15*, whose encoded protein is involved in clathrin-coated pit maturation, including invagination or budding [62]. Furthermore, EPS15 is involved in cell growth regulation, synaptic vesicle recycling and recruitment of alpha-adaptin [63]. Thus, the dysregulation of these genes could affect the normal vesicular trafficking of RPE cells, fundamental for many retinal processes, such as POS renewal and visual cycle intermediate regeneration, as well as avoiding AGE accumulation.

In order to reduce the deposition of such compounds, the ubiquitin-proteasome system represents an intracellular system that has to be fully functional. Three down-regulated *GLO1*-related genes, Ring Finger and FYVE Like Domain Containing E3 Ubiquitin Protein Ligase (*RFFL*), F-Box and WD Repeat Domain Containing 2 (*FBXW2*) and Cullin Associated and Neddylation Dissociated 1 (*CAND1*), play an important role inside this system. RFFL is an E3 ubiquitin-protein ligase that directly regulates cell migration through the mTORC2 complex and negatively regulates cell death downstream of death domain receptors in the extrinsic pathway of apoptosis [64,65]. FBXW2 is also an E3 ligase, which promotes the ubiquitylation and degradation of ß-catenin, up-regulated in the WNT/ß-catenin pathway as ca onsequence of inflammatory processes. FBXW2 functions as a substrate recognition receptor in the SCF (Skp1/Cullin/F-box) E3 ubiquitin ligase complex, involved in ubiquitin-dependent proteasomal degradation of cyclins and key metabolite enzymes [66], also promoted by CAND1. Additionally, CAND1 is able to regulate the tubular endoplasmic reticulum network, remodeling through elongation and retraction of tubules [67]. Thus, the down-expression of these three genes could impair the ubiquitin-proteasome system activity, favoring the accumulation of misfolded proteins and AGEs, inducing ER stress.

Interestingly, both FBXW2 and CAND1 play a role in glycolytic metabolism. Through the WNT/ß-catenin pathway, FBXW2 normally activates aerobic glycolysis (also called the Warburg effect), triggering the glycolytic enzymes Glut/PDK1/LDH-A/MCT-1 via PI3K/Akt/HIF-1α. This phenomenon allows the retinal cells to metabolize glucose and protect them against oxidative damage [68]. A similar metabolic effect is exerted by CAND1 by controlling the abundance of the glycolysis-promoting enzyme 6-Phosphofructo-2-Kinase/Fructose-2,6-Biphosphatase 3 (PFKFB3). This is a substrate for the SCF complex, acting during the S phase of cell cycle. Thus, the presence of PFKFB3 is tightly controlled to ensure the up-regulation of glycolysis at a specific point of the G1 phase [69]. Moreover, PFKFB3 maintains a high level of glycolytic intermediates (e.g., for synthesis of nonessential amino acids or hexosamine), whose regulation highlighted the importance of anaplerosis by glycolysis to overcome the restriction point of the G1 phase [70]. This scenario suggests that the down-expression of both *FBXW2* and *CAND1* could impair the glycolytic metabolism in RPE cells, altering the following cellular respiration process and inducing ROS production and cell death.

A weakening of glucose metabolism might also be determined by down-expression of Glutamine-Fructose-6-Phosphate Transaminase 1 (*GFPT1*), the first and rate-limiting enzyme of the hexosamine biosynthetic pathway (HBP), involved in ubiquitous glycosylation processes [71]. HBP activation results in the synthesis of UDP-N-acetylglucosamine (UDP-GlcNAc), a donor of N-acetylglucosamine (GlcNAc) for O-linked and N-linked glycosylation of a wide range of proteins, especially ones involved in signal transduction and the TGF-ß pathway [72]. The reduction of glycosylation seems to be compensated by over-expression of Polypeptide N-Acetylgalactosaminyltransferase 10 (*GALNT10*) which, however, only catalyzes the initial reaction in O-linked oligosaccharide biosynthesis, such as mucin-type oligosaccharides [73]. The opposite trend of *GFPT1* and *GALNT10* expression probably suggests a serious induced ER stress and cell–cell and cell–extracellular matrix attachment impairment, due to a decrease of GFPT1-related N-glycosylation, balanced by an increased O-glycosylation by GALNT10, which could be interpreted as a final attempt to guarantee mitochondrial respiration and redox homeostasis, as already demonstrated in retinal aging [74]. A defect in energy metabolism might also depend on dysregulation of SIK Family Kinase 3 (*SIK3*), which encodes for a specific kinase that positively regulates mTOR and CREB signaling, as well as cholesterol biosynthesis by coupling with retinoid metabolism and melanogenesis [75]. Thus, the observed down-expression of *SIK3* could decrease mitochondrial respiration and up-regulate removal of dysfunctional cellular components via autophagy, compromising cellular antioxidant mechanisms [76,77].

Intriguingly, a cluster of down-expressed *GLO1*-related genes is involved, directly or indirectly, in the regulation of translation machinery and cell survival. Decrease of Importin 7 (*IPO7*) could trigger p53 activation and p53-dependent growth arrest, also determining ribosomal biogenesis stress and nucleolar morphology changes [78]. Reduced levels of Mitochondrial Ribosomal Protein S33 (*MRPS33*) might damage mitochondrial protein synthesis [79]. MORC Family CW-Type Zinc Finger 4 (*MORC4*) and Microcephalin 1 (*MCPH1*) deregulation promotes apoptosis and arrests DNA damage repair [80,81]. Nuclear Factor I A (*NFIA*) down-regulation impairs mitotic exit and cell differentiation [82]. Cap Binding Complex Dependent Translation Initiation Factor (*CTIF*) down-expression alters mRNA and protein quality control, compromising the crosstalk between translation and the aggresome-autophagy pathway [83].

Finally, LMBR1 Domain Containing 1 (*LMBRD1*) down-expression showed by A2E-treated cells could impair the transport and metabolism of cobalamin, reducing the blood-to-retina supply of cyanocobalamin (vitamin B_12_) [84,85].

## 5. Conclusions

We report data obtained from whole transcriptome analysis performed on RPE cells, after exposure to A2E. Particularly, we focused on fold changes of genes sharing pathways with *GLO1*, comparing differential gene expression among control group cells and cultures at two different time points (3 and 6 h). We identified 22 *GLO1*-related genes that change their expression, and these are involved in a complex network of biochemical mechanisms that might be associated to RP onset and progression. Such pathways regarded microtubules and actin assembly, ubiquitin-proteasome activity, RE and Golgi integrity, vesicular trafficking, transcriptional and translational regulation, glycolytic metabolism control and glycosylation modifications.

However, obtained results could become more significant when an in vivo experiment will confirm what was observed in RPE cells, but with the wider scenario of the whole retina in a model organism. Moreover, other experimental procedures, such as immunoblots to validate obtained results at the protein level, will further clarify the relationship correlation among *GLO1* and these related genes here discussed.

Despite the aforementioned limitations, our results could represent an important step towards clarification of new *GLO1*-related molecular mechanisms behind RP etiopathogenesis.

## Figures and Tables

**Table 1:** Expression profiles of *GLO1* and its 22 best neighbors. Table shows expression profiles of *GLO1* and 22 genes that were the best *GLO1* neighbors from pathway analyses.

Name	Chr	Region	Database Object Name	Identifier	Due to Time (h)—Log Fold Change	Due to Time (h)—BH *p*-Value	3 vs. 0—Log Fold Change	3 vs. 0—BH *p*-Value	6 vs. 0—Log Fold Change	6 vs. 0—BH *p*-Value	6 vs. 3—Log Fold Change	6 vs. 3—BH *p*-Value
*GLO1*	6	complement(38675925..38703141)	Lactoylglutathione lyase	ENSG00000124767	−1.087494	0	−1.0874939	0	−0.92481018	0	0.16268372	0.034387327
*MRPS33*	7	complement(141002610..141015228)	Mitochondrial ribosomal protein S33	ENSG00000090263	−0.954493	1.0895 × 10^−15^	−0.9335623	1.054 × 10^−10^	−0.95449333	1.2368 × 10^−11^	−0.020931	0.009901671
*PTPN13*	4	86594315..86815171	Tyrosine-protein phosphatase non-receptor type 13	ENSG00000163629	−0.92721	4.0112 × 10^−10^	−0.5780328	0.0001813	−0.92721027	7.3951 × 10^−10^	−0.34917745	0.015625045
*MCPH1*	8	6406592..6648504	Microcephalin	ENSG00000147316	−0.893484	2.2186 × 10^−9^	−0.893484	2.92 × 10^−7^	−0.78536604	2.4954 × 10^−6^	0.10811791	0.007924506
*EPS15*	1	complement(51354263..51519328)	Epidermal growth factor receptor substrate 15	ENSG00000085832	−0.82938	0	−0.6739735	4.728 × 10^−12^	−0.82938046	0	−0.15540699	0.046473925
*FMNL2*	2	152335237..152649834	Formin-like protein 2	ENSG00000157827	−0.80498	0	−0.7772127	0	−0.80497977	0	−0.02776711	0.009594194
*CAND1*	12	67269281..67319951	Cullin-associated NEDD8-dissociated protein 1	ENSG00000111530	−0.794639	0	−0.6755685	0	−0.79463931	0	−0.11907082	0.053230752
*GFPT1*	2	complement(69319769..69387254)	Glutamine--fructose-6-phosphate aminotransferase [isomerizing] 1	ENSG00000198380	−0.744662	0	−0.7446622	0	−0.52883644	2.4312×10^−9^	0.21582578	0.012956862
*LMBRD1*	6	complement(69675802..69797111)	Probable lysosomal cobalamin transporter	ENSG00000168216	−0.709512	7.9331 × 10^−7^	−0.4893819	0.0012063	−0.70951157	8.9901 × 10^−7^	−0.22012963	0.047374267
*MORC4*	X	complement(106813871..107000244)	MORC family CW-type zinc finger protein 4	ENSG00000133131	−0.709235	8.711 × 10^−12^	−0.7092347	1.593 × 10^−9^	−0.57854773	2.7887 × 10^−7^	0.13068696	0.006919989
*ARHGAP21*	10	complement(24583609..24723668)	Rho GTPase-activating protein 21	ENSG00000107863	−0.528969	1.1901 × 10^−9^	−0.1276221	0.0259878	−0.52896918	4.8337 × 10^−10^	−0.40134704	5.73978 × 10^−5^
*IPO7*	11	9384622..9448126	Importin-7	ENSG00000205339	−0.5203	8.0463 × 10^−11^	−0.5203005	2.589 × 10^−11^	−0.28468508	0.00038239	0.23561538	0.020201474
*UBC*	12	complement(124911604..124917368)	Ubiquitin C	ENSG00000150991	−0.510574	1.0118 × 10^−12^	−0.2873309	8.702 × 10^−5^	−0.51057377	1.9955 × 10^−13^	−0.22324287	0.009893577
*NFIA*	1	60865259..61462793	Nuclear factor 1	ENSG00000162599	−0.482997	0.00653888	−0.0816926	0.0077167	−0.4829973	0.00283079	−0.40130466	0.058384899
*MYO18A*	17	complement(29071124..29180412)	Unconventional myosin-XVIIIa	ENSG00000196535	−0.475018	1.0417 × 10^−7^	0.30763414	0.0003741	−0.1673843	0.05367559	−0.47501844	2.43279 × 10^−7^
*SIK3*	11	complement(116843402..117098437)	Serine/threonine-protein kinase SIK3	ENSG00000160584	−0.469217	0.00033548	0.18183347	0.0175837	−0.28738387	0.01721478	−0.46921734	0.000380934
*FBXW2*	9	complement(120751978..120793412)	F-box/WD repeat-containing protein 2	ENSG00000119402	−0.351676	0.00050796	−0.115775	0.0361107	−0.35167614	0.00013619	−0.23590112	0.053634393
*SRGAP1*	12	63844293..64162221	SLIT-ROBO Rho GTPase activating protein 1, isoform CRA_a	ENSG00000196935	−0.262089	0.01446499	0.10500568	0.055908	−0.157083	0.0295634	−0.26208869	0.012864577
*CTIF*	18	48539046..48863217	CBP80/20-dependent translation initiation factor	ENSG00000134030	−0.147184	0.02306249	−0.1005342	0.0369055	−0.14718379	0.01161386	−0.0466496	0.007818362
*RFFL*	17	complement(35005990..35089319)	E3 ubiquitin-protein ligase rififylin	ENSG00000092871	−0.057893	0.00947048	−0.0578935	0.0084203	−0.0173127	0.00967554	0.04058077	0.009880382
*GALNT10*	5	154190730..154420984	Polypeptide N-acetylgalactosaminyltransferase	ENSG00000164574	0.1096478	0.04460188	0.07807127	0.0511209	0.10964776	0.02592625	0.03157648	0.008772447
*AUTS2*	7	69598919..70793068	Autism susceptibility gene 2 protein	ENSG00000158321	0.365981	0.00121048	0.36598102	0.0011642	0.29948291	0.00847909	−0.06649811	0.007682531
*ANKH*	5	complement(14704804..14871778)	Progressive ankylosis protein homolog	ENSG00000154122	0.5642544	8.6562 × 10^−5^	0.51466818	0.0008733	0.5642544	0.00013675	0.04958622	0.009202232

**Table 2:** Features of primers for qRT-PCR of ten selected genes. Table lists attributes of primers used for qRT-PCR validation of ten selected lncRNAs.

Gene Symbol	Gene ID (ENSEMBL)	Primer Forward (5′ → 3′)	Primer Reverse (5′ → 3′)	Length (bp)	TM (°C)
***CLIP1***	*ENSG00000130779*	*TGGCGTGGAGTTAGATGAGC*	*GGTGTAGTGGAAGGGAAGCC*	*138*	*62*
***SRSF5***	*ENSG00000100650*	*CCCGTGCCTGAGAAGAGC*	*TGCCACTGTCAACTGATCTGG*	*115*	*62*
***APOLD1***	*ENSG00000178878*	*GGACCAGATGCGAGAGATCC*	*CACGTGAGCCAAAGAAGACG*	*146*	*62*
***LYN***	*ENSG00000254087*	*AGTCTGATGTGTGGTCCTTTGG*	*GCTCATCTGGGCAGTTCTCC*	*147*	*62.5*
***RASGRP3***	*ENSG00000152689*	*ACTGTGCGGGATTTCTCTGG*	*CCCATGACCACTGCTCAAGG*	*146*	*62.5*
***ITGA4***	*ENSG00000115232*	*GAAAGAATCCCGGCCAGACG*	*GGCTGTCTGGAAAGTGTGACC*	*124*	*63.5*
***PDK4***	*ENSG00000004799*	*TTTCTACTCGGATGCTGATGAACC*	*GCATCTTGGACCACTGCTACC*	*121*	*63*
***SGK1***	*ENSG00000118515*	*AAATGTGAGTGGGCCCAACG*	*CTTGACGCTGGCTGTGACG*	*115*	*63.5*
***PIK3AP1***	*ENSG00000155629*	*GAAGCTGGGCATTGTCAACG*	*CTCTCTGTCTTCGGGTGATGC*	*143*	*63*
***SPP1***	*ENSG00000118785*	*CCGAGGTGATAGTGTGGTTTATGG*	*GGTGATGTCCTCGTCTGTAGC*	*97*	*63*

**Table 3:** Significant pathways involving the 22 *GLO1* most related neighbor genes. Table shows main significant pathways associated to 22 *GLO1* most related neighbor genes (bold and underlined), clustered in groups or unclustered.

**Function**	**Group Genes**
Asparagine N-linked glycosylation	ANK2|ANKRD28|CANX|CMAS|GBF1|**GFPT1**|GNPDA2|MAN1A2|MAN2A1|NAPG|NUS1|PGM3|SEC13|SEC23IP|ST6GALNAC5|TMED9|TUBA1C|**UBC**
Clathrin recruits PIK3C2A	**EPS15**|ITSN1|PIK3C2A|SCARB2|**UBC**
Glycolysis	AGL|ALDOA|ALDOC|ANK2|ANKRD28|BPGM|**CAND1**|CANX|CMAS|EP300|GALK2|GBF1|**GFPT1**|**GLO1**|GNPDA2|HOGA1|HOOK3|HSP90AA1|HSPA14|IGBP1|INSIG1|KIF22|KLHL12|MAN1A2|MAN2A1|**MYO18A**|NAPG|NUDT16|NUP107|NUS1|PGAM1|PGM3|PGP|PNP|PTGES3|RNASE4|SCARB2|SEC13|SEC23IP|SLC4A4|ST6GALNAC5|TMED9|TUBA1C|**UBC**|XRCC5
Glyoxylate and dicarboxylate metabolism	**GLO1**|GRHPR|HOGA1|MCEE|PGP
Golgi vesicle budding	ANKRD28|GBF1|INSIG1|KLHL12|MBTPS1|**MYO18A**|RNASE4|SCARB1|SEC13|SEC23IP|SP1|TMED9
Rho GTPase cycle	**ARHGAP21**|ARHGEF12|ARHGEF2|DLC1|FAM13A|ITSN1|RHOBTB1|**SRGAP1**
SMURFs ubiquitinate RUNX3	BMPR1B|EP300|FOS|GTF2H1|HTRA1|IFI16|IKBKG|INO80|MAPK7|MNAT1|NFRKB|RBL1|SMURF2|SP1|TGFBR3|**UBC**|XRCC5
Synthesis of PIPs at the plasma membrane	C1D|DLC1|EFNA5|FOS|KANK1|PIK3C2A|PIK3R1|PIP5K1B|**PTPN13**|RCC2|SBF2|**UBC**
Ubiquitin mediated proteolysis	CDC27|CUL7|RHOBTB1|SMURF2|**UBC**|UBE2E3|UBE2O|UBE2W
Unfolded Protein Response (UPR)	CUL7|EXOSC8|**GFPT1**|MBTPS1|SRPRB
Chromosomal region	ANTXR1|ASXL1|CCT4|CDC27|CEP152|DLC1|**FMNL2**|HSP90AA1|KIF22|KNTC1|NABP1|NDC80|NUF2|NUP107|PTGES3|RAD21|RCC2|SEC13|SKA1|SKA3|SSNA1|TAOK1|TBPL1|TEAD1|TERF2IP|THOC3|TINF2|TUBA1C|**UBC**|XRCC5|ZNF276|ZWILCH
Establishment of mitotic spindle orientation	ARHGEF2|ASXL1|**MCPH1**|NDC80
Regulation of lamellipodium organization	**AUTS2**|CD44|KANK1|NAA25
Translational termination	ANXA2|MRPS11|**MRPS33**|MRPS6|N6AMT1|RPS12|TXNDC5
**Function**	**Unclustered Gene**
Transcription factors	**MORC4**, **NFIA**
Ubiquitin protein ligase activity	**FBXW2**, **RFLL**
Positive regulation of mTOR signaling	**SIK3**
Nuclear import of proteins	**IPO7**
Translation initiation	**CTIF**
Catalysis of mucin-type oligosaccharides	**GALNT10**
PPi transport regulation	**ANKH**

## Supplementary Materials

The following are available online at https://www.mdpi.com/2076-3921/9/5/416/s1, Figure S1: Volcano plots based on *p*-values and fold-changes produced by Limma, Table S1: RNA-Seq differential expression analysis of the most dysregulated genes related to *GLO1*, Table S2: ClueGo and CluePedia pathway analysis of identified *GLO1*-related genes, Table S3: Pathways analysis of the 22 *GLO1* most related neighbor genes.
