# Supplementary material for: Discovery of GLO1 New Related Genes and Pathways by RNA-Seq on A2E-Stressed Retinal Epithelial Cells Could Improve Knowledge on Retinitis Pigmentosa
Source: Antioxidants (Basel). 2020 May 13;9(5):416. doi: 10.3390/antiox9050416 (PMC7278727; doi:10.3390/antiox9050416)
Supplement: Supplementary file 1 [file antioxidants-09-00416-s001.zip › Figure S1.pdf]

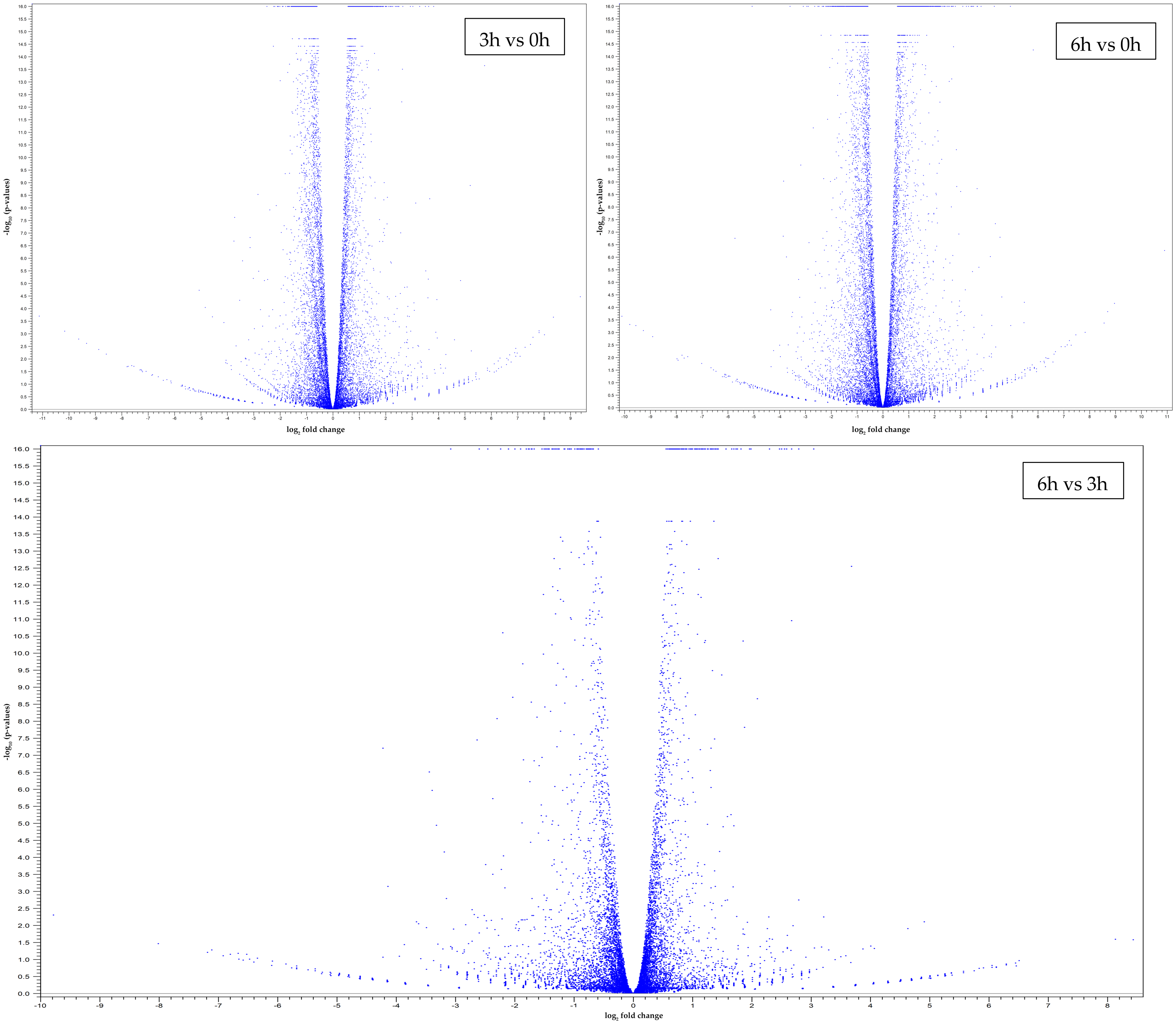

Figure S1. Volcano plots based on p-values and fold-changes produced by Limma. The volcano plot shows the relationship between the p-values of Limma included statistical test and the magnitude of the difference in expression values of the samples in the groups. On the y-axis, the $-log_{10}$ p-values are plotted, while the log_2 of the values in the “fold change” (or “Weighted fold change”) are plotted one the x-axis. The larger the difference in expression of a gene, the more extreme it's point will lie on the x-axis. The more significant the difference, the smaller the p-value and thus the higher the $-log_{10}(p)$ value. Thus, points for genes with highly significant differences lie high in the plot, and it is clear that many analyzed genes differ before and after applied treatment.
